# IUFRO Tree Biotechnology 2011: "From genomes to integration and delivery"

**DOI:** 10.1186/1753-6561-5-S7-A1

**Published:** 2011-09-13

**Authors:** Dario Grattapaglia

**Affiliations:** 1 EMBRAPA Genetic Resources and Biotechnology – Estação Parque Biológico, 70770-910, Brasilia, DF, Brazil; 2 Graduate Program in Genomics and Biotechnology - Universidade Catolica de Brasilia - SGAN 916 Modulo B, 70790-160 Brasilia, Brazil

## 

Forest trees have unquestionably entered the genomic era. The updated version of the *Populus* genome, the recently released *Eucalyptus grandis* genome and the concerted efforts towards the generation of genome sequences for spruces (*Picea sp.*) and pines (*Pinus sp.*) by several groups worldwide, are fueling a multitude of inter-disciplinary studies and applications in sustainable forest production and conservation. Time now calls for the integration of scientific fields with an increased sense of urgency for delivery of effective biotechnologies.

The IUFRO (International Union of Forestry Research Organizations) Tree Biotechnology biannual conference has established a solid tradition for over 20 years as the official meeting of the IUFRO working group 2.04.06 – Molecular biology of forest trees. This conference has convened scientists and foresters interested in the genetics, genomics, molecular biology and physiology of forest trees, and the application of this knowledge to tree improvement and conservation. The Tree Biotechnology Conference has undoubtedly been the premiere international forum where the most cutting edge research in tree biotechnology developed both in academia and industry is presented. “From genomes to integration and delivery”, this was the theme chosen for the 2011 edition of the IUFRO Tree Biotechnology Conference, first time to be held in South America. Our intention was to promote a more integrated and applied dialogue on tree biotechnology and genomics, beyond the mainstream discussion of the fundamental advances on the genetic mechanisms that underlie tree phenotypes.

In nine scientific sessions some of the current advances of genomics applied to forest conservation, tree physiology, stress response, molecular breeding, in vitro and propagation technologies, wood development and genetically modified (GM) trees were highlighted. With 340 registered participants, the Conference brought to Brazil most of the world’s brain power in forest tree genomics and biotechnology. An outstanding team of international scientists shared their results and visions on the present and future of this fast moving area of forest science, while a brilliant group of young scientist and students delivered a very energetic and diverse collection of high-quality scientific presentations. Forty two countries were represented at the Conference with almost 100 different laboratories from tens of Universities, research institutions and private companies.

During the seven days of the Conference 26 invited lectures, 63 oral and 185 poster presentations were delivered, totaling 274 papers made available as extended abstracts into this BMC Proceedings supplement. The special workshop on the hot topic of “Genomic Selection in tree breeding” and the several reports on whole-genome studies, made this conference edition inaugurate a deliberate effort towards a better integration between the quantitative genomics, the “single-gene” and the system biology approaches to more efficiently unravel the complex relationships between genotypes and phenotypes in forest trees. A field trip to the forest plantations, nurseries and mill of VERACEL Cellulose was a definite highlight and a welcome break from the scientific sessions, providing an overview of some of the advances and challenges facing the translation of research into plantation forestry.

In closing this introductory statement, acknowledgements are due to the outstanding financial support provided by the competitive grants of the Brazilian Ministry of Science and Technology through the National Research Council (CNPq) and the Ministry of Education through its agency for graduate studies (CAPES). Major support was also provided by EMBRAPA (Brazilian Corporation of Agricultural Research), and VERACEL Cellulose, the host organizations, together with an exceptional suite of private sponsors. Besides the organizations that backed this conference and an active Scientific Committee involved in abstract review a number of people were involved in the organization and logistics. The conference would not have been possible without the valuable contributions of all these players.

Given the rewarding feedback received after the Conference, the original goal of providing an exceptional mix of science, social activities and field exploration in a relaxed atmosphere was truly accomplished. The IUFRO Tree Biotechnology Conference 2011 made a significant contribution to advance the forest biotechnology research community one step ahead on the challenging task of moving from gene and genome discoveries to the delivery of valuable technologies into sustainable forestry.

